# Comparison of Low and Moderate-Complexity Enrollees in the GUIDE Dementia Care Model

**DOI:** 10.1093/geroni/igaf122.2849

**Published:** 2025-12-31

**Authors:** Karina Berg, Richard Fortinsky, Julie Robison, Shawn Ladda, Michelle Wyman, Wendy Martinson, Eleanor Miller

**Affiliations:** UConn Health, Farmington, Connecticut, United States; University of Connecticut, Farmington, Connecticut, United States; University of Connecticut, Farmington, Connecticut, United States; UConn Health, Farmington, Connecticut, United States; UConn Health, Farmington, Connecticut, United States; UConn Health, Farmington, Connecticut, United States; UConn Health, Farmington, Connecticut, United States

## Abstract

In July 2024, the Center for Medicare and Medicaid Innovation (CMMI) launched the Guiding an Improved Dementia Experience (GUIDE) model in clinical settings. In GUIDE, people living with dementia (PLWD)-caregiver dyads are assigned to complexity tiers based on dementia severity level. Low-tier dyads receive less frequent outreach, are not eligible for respite funds, and garner a lower monthly payment from Medicare. Our clinical experience suggested that low-tier PLWD exhibit similar symptoms, and their caregivers face similar unmet needs as those in higher tiers. To empirically test our observations, we compared PLWD behaviors, caregiver burden, and unmet service needs between low-tier and higher-tier dyads. Data collected at enrollment included presence/absence of six PLWD behaviors (e.g.,irritability, paranoia); caregiver burden (Zarit Index); and nine caregiver-reported needs (e.g.,in-home services, stress management). Low vs. moderate tier dyads were compared using t-tests; the few high-tier dyads were excluded. PLWD (n = 61) were 62% female with mean(sd) age 83(9) years. Caregivers (n = 61) were 72% female with mean(sd) age 57(12) years, 47% were children, 44% spouses/partners. Thirty-two dyads (52.5%) were deemed low-tier by CMMI. Mean number of behaviors for low vs moderate-tier PLWD was 2.2 vs. 2.7, respectively (p = 0.16), mean burden scores were 35.5 vs. 34.1 (p = 0.36), and mean number of service needs was 3.4 vs. 3.8 (p = 0.26). Results indicate that low and moderate-tier dyads have similar levels of PLWD behaviors, caregiver burden, and unmet needs, suggesting that criteria for low-tier complexity should consider factors beyond dementia severity to better align GUIDE service delivery and reimbursement with care needs.

